# Analysis of clinical parameters and echocardiography as predictors of fatal pediatric myocarditis

**DOI:** 10.1371/journal.pone.0214087

**Published:** 2019-03-20

**Authors:** Yi-Jung Chang, Hsiang-Ju Hsiao, Shao-Hsuan Hsia, Jainn-Jim Lin, Mao-Sheng Hwang, Hung-Tao Chung, Chyi-Liang Chen, Yhu-Chering Huang, Ming-Han Tsai

**Affiliations:** 1 Department of Pediatrics, Chang Gung Children’s Hospital, Taoyuan, Taiwan; 2 Chang Gung University College of Medicine, Taoyuan, Taiwan; 3 Molecular Infectious Disease Research Center, Chang Gung Memorial Hospital, Taoyuan, Taiwan; 4 Department of Pediatrics, Chang Gung Memorial Hospital, Keelung, Taiwan; University of Messina, ITALY

## Abstract

Pediatric myocarditis symptoms can be mild or as extreme as sudden cardiac arrest. Early identification of the severity of illness and timely provision of critical care is helpful; however, the risk factors associated with mortality remain unclear and controversial. We undertook a retrospective review of the medical records of pediatric patients with myocarditis in a tertiary care referral hospital for over 12 years to identify the predictive factors of mortality. Demographics, presentation, laboratory test results, echocardiography findings, and treatment outcomes were obtained. Regression analyses revealed the clinical parameters for predicting mortality. During the 12-year period, 94 patients with myocarditis were included. Of these, 16 (17%) patients died, with 12 succumbing in the first 72 hours after admission. Fatal cases more commonly presented with arrhythmia, hypotension, acidosis, gastrointestinal symptoms, decreased left ventricular ejection fraction, and elevated isoenzyme of creatine kinase and troponin I levels than nonfatal cases. In multivariate analysis, troponin I > 45 ng/mL and left ventricular ejection fraction < 42% were significantly associated with mortality. Pediatric myocarditis had a high mortality rate, much of which was concentrated in the first 72 hours after hospitalization. Children with very high troponin levels or reduced ejection fraction in the first 24 hours were at higher risk of mortality, and targeting these individuals for more intensive therapies may be warranted.

## Introduction

Myocarditis is a potentially life-threatening inflammatory disorder of the myocardium, which is difficult to diagnose due to its nonspecific and inconsistent clinical presentation [1]. Signs and symptoms of acute myocarditis in children vary from mild flu-like illness to fatal cardiogenic shock. Some patients may rapidly deteriorate from apparent good health and develop serious complications such as ventricular arrhythmias, cardiogenic shock, or cardiac arrest, which often occur unexpectedly soon after hospitalization [2]. The mortality rates for infants and children with myocarditis can be as high as 75% and 25%, respectively [3–5], and therefore, identifying the predictors of death among pediatric myocarditis patients is imperative. Several studies have examined the diagnosis and treatment of acute myocarditis within pediatric groups [6–11]; however, few reports have specifically focused on evaluating the severity of the illness [9,11]. It remains unclear as to whether these individuals had more fulminant infections, more profound immune or other clinical responses, more complications or multiorgan system involvement at the time of diagnosis, later presentation or more advanced disease at presentation. Furthermore, the predictors of mortality in pediatric acute myocarditis are yet to be established. Clarifying the risk factors for mortality in children with myocarditis may facilitate effective intensive care and timely provision of circulatory support before circulatory collapse occurs. To address this gap in current knowledge, we aimed to identify the risk factors for mortality in patients with acute myocarditis in order to guide higher-risk patients to higher levels of care.

## Methods

### Study population and data collection

**Fig 1:**
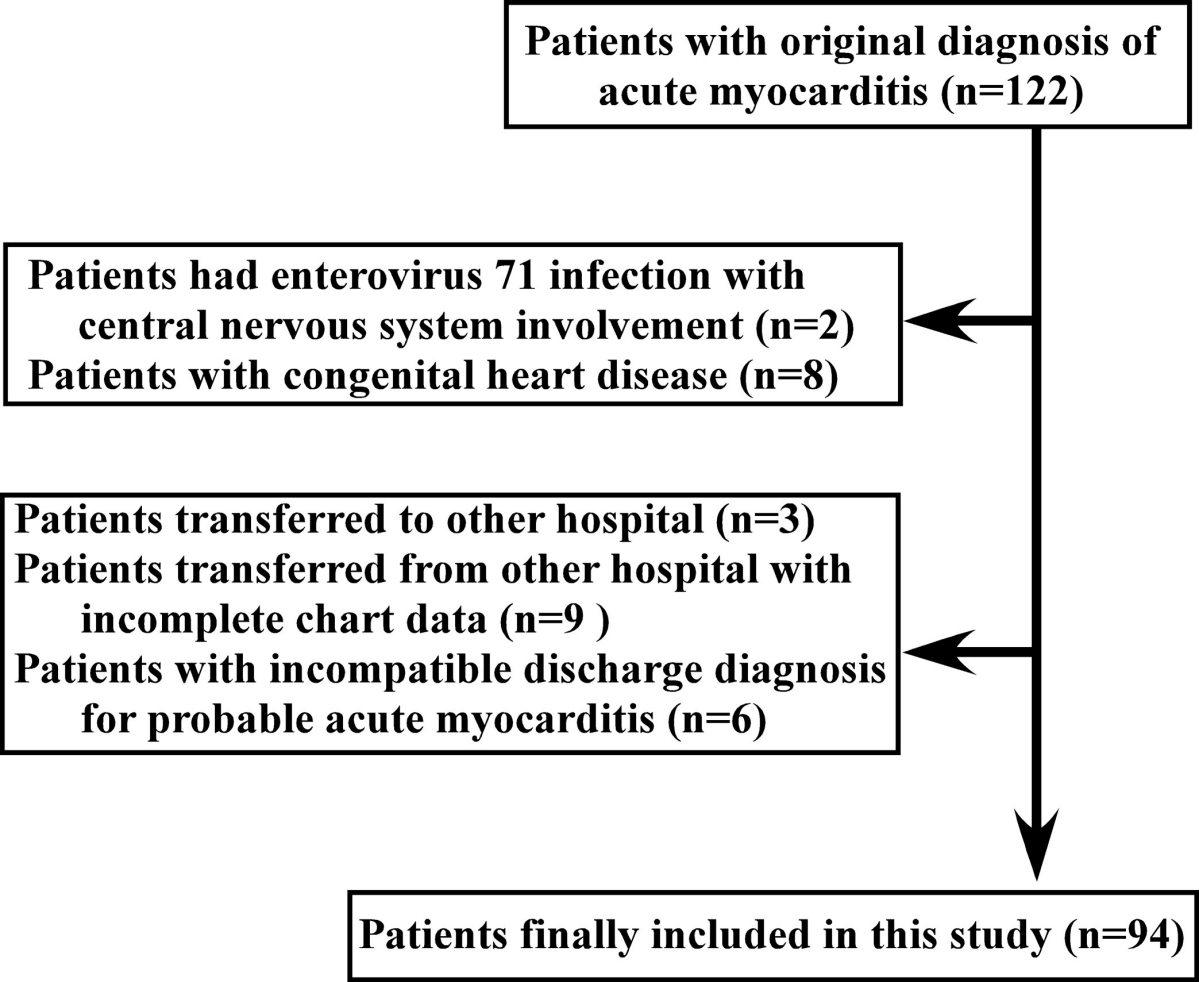
Flow diagram for selection of myocarditis cases.

We retrospectively reviewed the charts of patients (age <18 years) who initially visited the pediatric emergency department of Chang Gung Memorial Hospital, a tertiary care referral hospital, and had a discharge diagnosis of acute myocarditis. Data were obtained from January 2004 to December 2015. Patients were identified through hospital database searches for all children who had an International Classification of Diseases, Ninth Revision (ICD 9) and a clinical modification discharge diagnostic code corresponding to myocarditis. The identified patients were further screened and selected, resulting in a cohort of 94 patients (Fig 1).

All selected cases had a final diagnosis of acute myocarditis and subsequent adjudication by attending pediatric cardiologists who confirmed the clinical diagnosis at the time of hospital discharge. Acute myocarditis was clinically diagnosed on the following basis: (i) symptoms and physical examinations of acute heart failure and rapid deterioration; (ii) history of flu-like illness within the preceding 2 weeks; (iii) cardiomegaly (cardiothoracic ratio > 0.5) on the chest radiograph, or impaired heart contractility on echocardiography (ejection fraction <55%); and (iv) elevation of cardiac enzyme levels [troponin I (TnI) >0.1 ng/mL] or creatine kinase [CK-MB >6.3 ng/mL] [2,12–15].

We excluded children with congenital heart disease, history of cardiomyopathy, acute cardiopulmonary failure with fulminant enterovirus 71 infections with central nervous system involvement, as indicated by brain magnetic resonance imaging [16], or those deemed to have insufficient documentation in their medical records for analysis. Demographic characteristics, clinical features, electrocardiogram (ECG) and echocardiography findings, treatment modalities, and laboratory variables and outcomes were retrieved from electronic medical records for each patient. Patients were followed through the course of the study period from 2004 to 2017 for late outcomes. This study was approved by the institutional review board of Chang Gung Memorial Hospital.

### Statistical analyses

To identify the predictors for mortality in pediatric myocarditis, we classified the patients into two groups according to their outcomes: patients with fatal status (fatal group) and those with nonfatal status (survivors’ group). We compared and analyzed the selected variables between the two groups. We arbitrarily classified the clinical presentations into the following five subgroups for analysis: respiratory (shortness of breath), gastrointestinal (GI) (abdominal pain or vomiting), cardiac (chest pain or palpitations), hypoperfusion (syncope or seizure), and fever [2,10].

Distributions of variables are presented as means ± standard deviation or the number of patients and percentage. Univariate analyses were performed, where applicable, with the chi-squared test or Fisher’s exact test used for categorical variables and the student’s t-test used for numerical variables. Receiver-operating characteristic (ROC) curve analysis of the statistical significance of numerical variables was performed, and the Youden index was calculated to best evaluate the cutoff point for the diagnosis of fatal myocarditis. Multivariate analyses were conducted using logistic regression analyses; fatal myocarditis was the outcome of interest and thus identified as the independent variable, and all other significant variables included in the univariate or ROC analyses were identified as dependent variables. SPSS for Windows version 22.0 (Statistical Product and Service Solutions, SPSS Inc., Chicago, IL, USA) was used for all data analysis. All statistical tests were evaluated at the 5% significance level.

## Results

### Patients’ clinical characteristics, manifestations, and laboratory findings

A total of 94 patients (51 boys and 43 girls) with myocarditis were identified, with a median age of 10 years at presentation. In total, 74 (78.7%) met all 4 criteria. The other 20 were diagnosed by a pediatric cardiologist at the time of discharge, including 18 had normal LVEF (EF≧55%) but with elevated median troponin I level of 5.852 ng/mL (arrange 0.116–23.251 ng/mL), one had normal troponin I level (0.014 ng/mL) but with impaired LVEF (EF:54%), and the other one had normal troponin I level (0.010 ng/mL) but elevated CK-MB level (17 ng/mL). Of the 94 patients, 45 (47.8%) were transferred from an outside hospital emergency department. During admission, 16 patients (17%) died (fatal group) within this hospital admission, and 78 patients remained alive (survivors’ group). Among the fatal group, 12 patients (75%) died within the first 72 hours of hospital admission, with five (41.6%) undergoing cardiopulmonary resuscitation (CPR) within the first 3 hours of hospitalization.

The clinical characteristics of the 94 patients are summarized in Table 1. Most cases were symptomatic for 2–4 days. That is, circulatory collapse occurred mostly in the first week. Cardiac symptoms were the most common complaint upon admission (38.2%), followed by fever (27.6%) and gastrointestinal symptoms (23.4%). Twenty-nine patients (30.8%) were affected with virus infections. Coxsackie virus was the most common virus associated with myocarditis (40.6%). Forty-eight patients (51.1%) had hypotension upon admission.

**Table 1:** Characteristics of 94 patients with pediatric myocarditis on admission.

Variables	Fatal group	Survivors group	Total	*p*
	(N = 16) n (%)	(N = 78) n (%)	(N = 94) n (%)	
Male gender	7 (44.0)	44 (56.4)	51 (54.2)	0.354
Age (years) (mean, SD)	9 ± 5.1	10.3 ± 5.3	10.1 ±5.3	0.36
Hospital stay (days)	14.8 ± 19	12.7 ± 11.6	13 ± 13	0.666
Duration of symptoms before admission (days)	3.7 ± 4.3	1.9 ± 2.2	2.2 ± 2.7	0.145
Gastrointestinal symptoms	10 (62.5)	12 (15.3)	22 (23.4)	< 0.001
Respiratory symptoms	4 (25.0)	10 (12.8)	14 (14.8)	0.249
Cardiac symptoms	1 (6.2)	35 (44.8)	36 (38.2)	0.004
Fever	7 (43.7)	19 (24.3)	26 (27.6)	0.132
Hypoperfusion	2 (12.5)	15 (19.2)	17 (18.0)	0.728
ECMO usage	8 (50.0)	13 (16.6)	21 (22.3)	0.007

ECMO = extracorporeal membrane oxygenation

We provided extracorporeal membrane oxygenation (ECMO) to 21 patients (22.3%). Of the non-survivors, half (8/16) did not receive ECMO support. Among the eight non-survivors with ECMO support, most occurred before 2012, with complications of ECMO such as intracranial hemorrhages. Apart from complications of ECMO, the most common cause of death in non-survivors was heart failure with metabolic acidosis and some were complicated with hypoxic ischemic encephalopathy or multiple organ failure. Arrhythmias resulting in hemodynamic compromise (complete atrioventricular [AV] block, ventricular tachycardia [VT])], or ventricular fibrillation [VF]) were recorded in 29 children (30.9%), and pacemakers were implanted in 15 children (15.9%). Eighty-five (90.4%) patients were admitted to the intensive care unit (ICU). The average hospitalization time was 13.2±13.1 days. Among the cardiac markers examined, troponin I, creatine phosphokinase-MB (CPK-MB), and B-type natriuretic peptides (BNP) were elevated in a large number of patients at the time of admission (94.6%, 81.2%, and 57.6%, respectively). The results of univariate analyses between the fatal and survivors’ groups are shown in Table 1.

### Clinical outcomes and predictors of prognosis

Age, sex, and the duration of symptoms before admission were not statistically different between the two groups. Patients in the fatal group presented significantly more frequently with gastrointestinal symptoms, less cardiac symptoms, and more ECMO usage than the survivors’ group. Laboratory characteristics and cardiac examination findings of the patients are illustrated in Table 2. Patients in the fatal group were significantly more likely to have hypotension, arrhythmia, metabolic acidosis, lower left ventricular ejection fraction (LVEF), and elevated troponin I and CK-MB levels than those in the survivors’ group.

**Table 2:** Comparison of laboratory characteristics and survey findings of 94 patients with pediatric myocarditis.

		Univariate analysis		Multivariate analysis
Characteristics	Fatal group	Survivors group	*p*	*p*
	(N = 16) n (%)	(N = 78) n (%)		
Arrhythmia	11 (68.8)	21 (26.9)	0.032	0.447
Hypotension	16 (100)	32 (41.0)	0.001	0.997
LVEF (%) on admission*	37.3 ± 15.6	58.8 ± 15.3	< 0.001	
< 42%	12 (87.5)	13 (17.3%)	< 0.001	0.036
Nadir LVEF (%)*	31.7 ± 16.3	56.1±16.5	< 0.001	
Acidosis (PH)*	7.23 ± 0.24	7.38 ± 0.12	< 0.001	0.598
Troponin-I (ng/mL)*	53.1 ± 50.7	14.4 ± 23.8	< 0.002	
≥ 45 (ng/mL)	10 (62.5)	6 (7.6)	< 0.001	0.033
CPK (U/L)*	2925 ± 1813	1358 ± 1813	0.92	
CK-MB (ng/mL)*	236 ± 135.8	63 ± 86.1	0.002	0.577
BNP (pg/mL)*	1431 ± 1978	886 ± 1467	0.472	
WBC (×10^9^/L)*	12.5 ± 5.8	11.1 ± 5.9	0.393	
CRP (mg/L)*	26.3 ± 40.1	33.6 ± 48.9	0.583	
Blood sugar (mg/dL)*	123.6 ± 62.8	124 ± 53.7	0.985	
AST (U/L)*	520.3 ± 630.7	342.4 ± 1145.1	0.55	
GI symptoms	10 (62.5)	12 (15.3)	< 0.001	0.535

*mean ± standard deviation

LVEF = left ventricular ejection fraction; CPK = creatine phosphokinase; CK-MB creatine phosphokinase-MB; BNP = brain natriuretic peptide; WBC = white blood count; CRP = C-reactive protein; AST = aspartate aminotransferase; GI symptoms = Gastrointestinal symptoms

**Fig 2:**
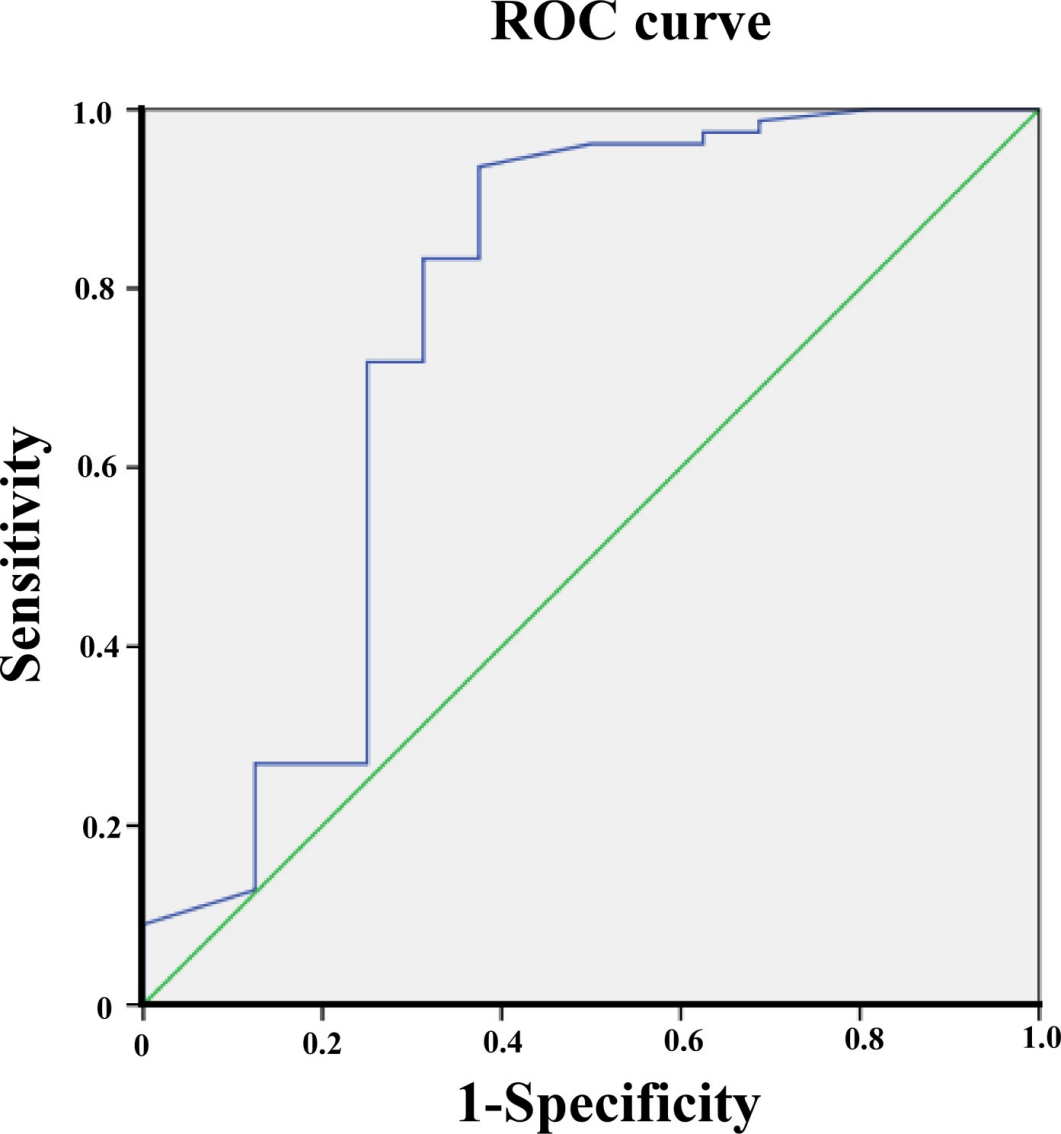
Receiver operating characteristic curve for initial serum troponin-I in predicting the mortality of pediatric myocarditis. The area under the curve was 0.76. The best cutoff value for serum troponin-I was 45 ng/mL (sensitivity, 0.62; specificity, 0.91).

The ROC analysis revealed that the area under the ROC (AUROC) for the initial LVEF in predicting mortality was 0.82 and the best cutoff value of the initial LVEF for predicting mortality was 42% (sensitivity: 85.7%, specificity: 82.8%). AUROC for the initial serum troponin I level in predicting mortality was found to be 0.76 and the best cutoff value of the initial troponin I level for predicting mortality was 45 ng/ml (sensitivity: 62.5%, specificity: 91%) (Fig 2). Gastrointestinal symptoms, hypotension, arrhythmia, LVEF, metabolic acidosis, and levels of troponin I and CK-MB were further considered in a multivariate logistic regression analysis. Ejection fraction (EF) < 42% and troponin I > 45 ng/mL were identified to have be most significant associations with mortality (Table 3). When troponin I was >45 ng/mL, the patient was prone to, and more frequently demonstrated, hypotension (*p* = 0.004, OR = 5.9, 95% CI 1.92 to 42.5) and arrhythmia (*p* < 0.001, OR = 8.47, 95% CI 3.07 to 37.6). When LVEF was <42%, significantly more patients had hypotension (*p* = 0.001, OR = 6, 95% CI 2.07 to 18.76). If patients presented with both troponin I > 45 ng/ml and EF < 42%, the positive predictive values (PPV) was 100% and the negative predictive values (NPV) was 90.4% for a fatal outcome.

**Table 3:** Sensitivity, specificity, and odd ratios of predictor of fatal pediatric myocarditis.

Finding	Sensitivity (%)	Specificity (%)	Odds ratio (95% confidence interval)
Troponin-I > 45 ng/mL	62.5	91	31.8 (2.1–479.4)
Ejection fraction < 42%	85.7	82.8	20.7 (1.3–315.1)
Both findings	57.1	100	197.4 (10.2–3820.5)

## Discussion

Pediatric myocarditis is relatively uncommon as a final hospital diagnosis; however, its occurrence is associated with high mortality rates, particularly in the first 72 hours. Our study identified highly variable presentations of pediatric myocarditis, from mild to critical and rapidly-progressive; therefore clinical courses were found to vary between patients. Two independent predictors of in-hospital mortality were identified in the current study which may help clinicians triage these individuals to more rapid intensive care and administer more intensive therapies such as circulatory support.

Acute myocarditis may present a clinical course of great contrast, with critical illness at presentation, however, a complete recovery is possible. In our study, the mortality rate of around 15% was consistent with previous pediatric reports which have ranged from 7.2% to 27.2% [17–20]. To the best of our knowledge, this is the first study which has found the risk period of circulatory collapse to be most prevalent within the first week of symptoms. If death occurred, it was usually within the first 72 hours of hospitalization, and approximately one-third of cases required CPR in the first 3 hours following admission. This finding is similar to that of Matitiau et al who noted that all four deaths of a 24 case series occurred within hours of presentation to the hospital [21]. If children survive the initial myocarditis, they usually have favorable prognosis. Lee et al. reviewed 35 cases of myocarditis, with 66% of the patients studied admitted to the ICU [17]. In a study of children with acute fulminant myocarditis in France, all of the eleven patients studied required ICU admission [22]. In our series, almost all patients were admitted to the ICU, but only half of them were serious with hemodynamic compromise. This may due to the lack of clarity for first line physicians as to whether these individuals had more fulminant decompensation with this disease at the time of diagnosis. Therefore, the use of serum markers has drawn increased attention to accuracy in the early diagnosis and evaluation of myocarditis [13,23–25].

Troponin has been previously well established as a diagnostic marker for myocarditis; however, limited data are available on its prognostic significance. The present study revealed that fatal group patients had significantly higher levels of troponin I than those in the survivors’ group. Previous single-center studies of children with myocarditis have not established consistent correlations between troponin elevation and poor outcomes [26–28]. Recent studies concluded that abnormal troponin in the first 72 hours after hospitalization for myocarditis was associated with the use of ECMO. Furthermore, there is very little knowledge regarding the association between hemodynamic compromised dysrhythmias and prognostic values of troponin I levels in pediatric myocarditis. The current study findings indicated that cardiac troponin I concentrations carry important prognostic information, mainly with respect to mortality and/or hemodynamic compromised dysrhythmias. Our study showed that troponin I > 45 ng/ml was significantly associated with an eight time increase in the odds of hemodynamic compromised dysrhythmias.

Arrhythmia has been identified as a risk factor for pediatric myocarditis. In a previous study, the presence of ventricular tachyarrhythmia and bradyarrhythmia were associated with an increase in requiring ECMO therapy. The incidence of hemodynamically significant arrhythmias on initial presentation was 30.9% in this series, which was consistent with rates observed in previous studies [11,29]. By identifying the high risk of mortality and arrhythmia in patients with myocarditis, pinpointing those with troponin I > 45 ng/ml, and ensuring timely introduction of critical care and ECMO in the clinic where appropriate, the survival rates of patients with acute myocarditis may be increased.

In agreement with previous studies [30,31], depressed LVEF was found to be significantly correlated with poor outcomes. Echocardiography is a key component for the evaluation of myocardial severity and to rule out other causes of heart failure [32,33]. In an earlier study, Butts et al. found lower echo shortening fraction to be a predictor of mortality in children with myocarditis [34]. Similarly, Magnani et al. used a multivariate predictive model and identified presentations with syncope, bundle branch block, or an EF < 40% as significant predictors of increased risk of death or transplantation [35]. These findings were consistent with the case series by Herskowitz [36]. Our study found an EF <42% to be significantly associated with a 20 times increase in the odds of hypotension. Previous studies have noted hypotension to predict a poor prognosis for pediatric myocarditis [10]. Given the potential for acute decompensation with myocarditis, it is paramount that clinicians are aware of this relatively common presentation. Some pediatric patients with myocarditis do not present with symptoms directly linked to the cardiovascular system [23]. In our univariate analysis, a history of gastrointestinal symptoms conferred a greater risk of mortality. Heart failure symptoms frequently present as abdominal pathology in pediatric patients [2]. Pediatric practitioners should be aware of gastrointestinal symptoms as common misdiagnosis with excessive intravenous fluids at emergency departments, which may exacerbate the heart failure.

Strengths of this study include the comprehensive approach and analysis of clinical parameters to predictors of outcome in pediatric myocarditis at our institution, the median age of the patients as early teenage years, the relatively common presentations of patients who had abdominal symptoms, the large proportion of patients in whom circulatory collapse occurred in the first week of symptoms and the mortality rates within the first 72 hours of hospitalization. Although this study represents a large pediatric cohort of myocarditis patients, there were some limitations. Firstly, the retrospective nature of the study, as inevitably, there are some missing records or laboratory findings in the medical charts. Infants with sudden infant death syndrome or child with out-of-hospital cardiac arrest may have incompletely study for myocarditis when resuscitation, which may cause underdiagnosis of fatal cases. Secondly, it was conducted within a single academic medical center with a large referral population. A population-based study is needed for the general population. Third, in contrast to other institutions, confirmed endomyocardial biopsy or magnetic resonance is not a standardized component for the evaluation of suspected cardiomyopathy at our center. The definition of definite acute myocarditis was based on endomyocardial biopsy results according to the Dallas criteria [37]. Meanwhile, two (2.12%) patients with normal troponin I level but having abnormal LVEF or CK-MB level were included in this study, which may affect the results. The lack of unequivocal diagnostic criteria allows one to question the diagnosis of myocarditis; this is not unique to this study [2,10]. Probable acute myocarditis was defined as acute myocarditis that was clinically determined by pediatric cardiologist based on the patient’s history, physical examination, and results of laboratory investigations (in the absence of endomyocardial biopsy). Finally, we defined all of the cases in our study as probable acute myocarditis.

## Conclusions

Most instances of mortality due to pediatric myocarditis occurred in the first 72 hours after admission. In the first 24 hours of admission, levels of troponin I may be a useful marker for predicting the severity of myocarditis. LVEF is also important for identifying the prognostic outcome. We determined that troponin I > 45 ng/mL and LVEF < 42% predict mortality in pediatric myocarditis. Due to the fulminant course, earlier recognition of patients who will rapidly progress to refractory cardiac failure is warranted to improve clinical outcome.

## Abbreviations

LVEF: left ventricular ejection fraction
TnI: troponin I
CK-MB: isoenzyme of creatine kinase
ECG: electrocardiogram
GI: gastrointestinal
ROC: receiver-operating characteristic
CPR: cardiopulmonary resuscitation
ECMO: extracorporeal membrane oxygenation
AV: atrioventricular
VT: ventricular tachycardia
VF: ventricular fibrillation
ICU: intensive care unit
CPK-MB: Creatine phosphokinase-MB
BNP: B-type natriuretic peptides
AUROC: area under the ROC
EF: Ejection fraction
PPV: positive predictive values
NPV: negative predictive values
AST: aspartate transaminase
ALT: alanine transaminase
